# Hemodynamic management of cardiogenic shock in the intensive care unit

**DOI:** 10.1016/j.healun.2024.03.009

**Published:** 2024-03-20

**Authors:** Hoong Sern Lim, José González-Costello, Jan Belohlavek, Elric Zweck, Vanessa Blumer, Benedikt Schrage, Thomas C. Hanff

**Affiliations:** a Institute of Cardiovascular Sciences, University of Birmingham, Birmingham, UK; b University Hospitals Birmingham NHS Foundation Trust, Birmingham, UK; c Advanced Heart Failure and Heart Transplant Unit, Department of Cardiology, Hospital Universitari de Bellvitge, BIOHEART-Cardiovascular Diseases Research Group, Bellvitge Biomedical Research Institute (IDIBELL), L’Hospitalet de Llobregat, Barcelona, Spain; d Department of Clinical Sciences, School of Medicine, Universitat de Barcelona, Barcelona, Spain; e Ciber Cardiovascular (CIBERCV), Instituto Salud Carlos III, Madrid, Spain; f 2nd Department of Medicine-Department of Cardiovascular Medicine, First Faculty of Medicine, Charles University in Prague and General University Hospital, Prague, Czech Republic; g Institute of Heart Diseases, Wroclaw Medical University, Wroclaw, Poland; h Department of Cardiology, Pulmonology and Vascular Medicine, University Hospital Duesseldorf, Duesseldorf, Germany; i Inova Schar Heart and Vascular Institute, Inova Fairfax Medical Campus, Falls Church, Virginia; j University Heart and Vascular Centre Hamburg, German Centre for Cardiovascular Research, Partner Site Hamburg/Kiel/Lübeck, Hamburg, Germany; k Division of Cardiovascular Medicine, University of Utah School of Medicine, Salt Lake City, Utah.

**Keywords:** cardiogenic shock, mechanical circulatory support, hemodynamics, inotropes, heart transplantation, left ventricular assist devices

## Abstract

Hemodynamic derangements are defining features of cardiogenic shock. Randomized clinical trials have examined the efficacy of various therapeutic interventions, from percutaneous coronary intervention to inotropes and mechanical circulatory support (MCS). However, hemodynamic management in cardiogenic shock has not been well-studied. This State-of-the-Art review will provide a framework for hemodynamic management in cardiogenic shock, including a description of the 4 therapeutic phases from initial ‘Rescue’ to ‘Optimization’, ‘Stabilization’ and ‘de-Escalation or Exit therapy’ (R-O-S-E), phenotyping and phenotype-guided tailoring of pharmacological and MCS support, to achieve hemodynamic and therapeutic goals. Finally, the premises that form the basis for clinical management and the hypotheses for randomized controlled trials will be discussed, with a view to the future direction of cardiogenic shock.

The prevalence of cardiogenic shock (CS) has been variably reported, typically accounting for about 15% of intensive care unit admissions, with a increasing trend of CS unrelated to acute myocardial infaraction (AMI).^1^ Short-term mortality is high at 50–60%, with most deaths within 30 days related to cardiac causes.^2^ The assessment, manipulation and targeting of (macro) circulatory parameters are integral to the management of CS with the general presumption that improving global hemodynamic parameters would translate into improvements in tissue perfusion. Advances in mechanical circulatory support (MCS) have brought sharp focus to the hemodynamic management of patients with CS. In this State-of-the-Art review will provide a framework for a more nuanced hemodynamic management in CS and examine the results from key randomized controlled trials (RCTs).

## Therapeutic goals in cardiogenic shock

We have been well-servedF by numerous RCTs in CS (Supplementary Material), but these trials have offered little guidance on hemodynamic management of CS. Hemodynamic management in the RCTs of CS were universally delegated to the treating clinicians’ discretion, often without an over-arching therapeutic strategy or explicit therapeutic goals. A framework for hemodynamic management for CS with the combination of pharmacological (vasoactive drugs) and MCS (termed pharmaco-MCS) should consider (1) the evolution of CS (therapeutic phases); and (2) the therapeutic goals.

**Figure 1:**
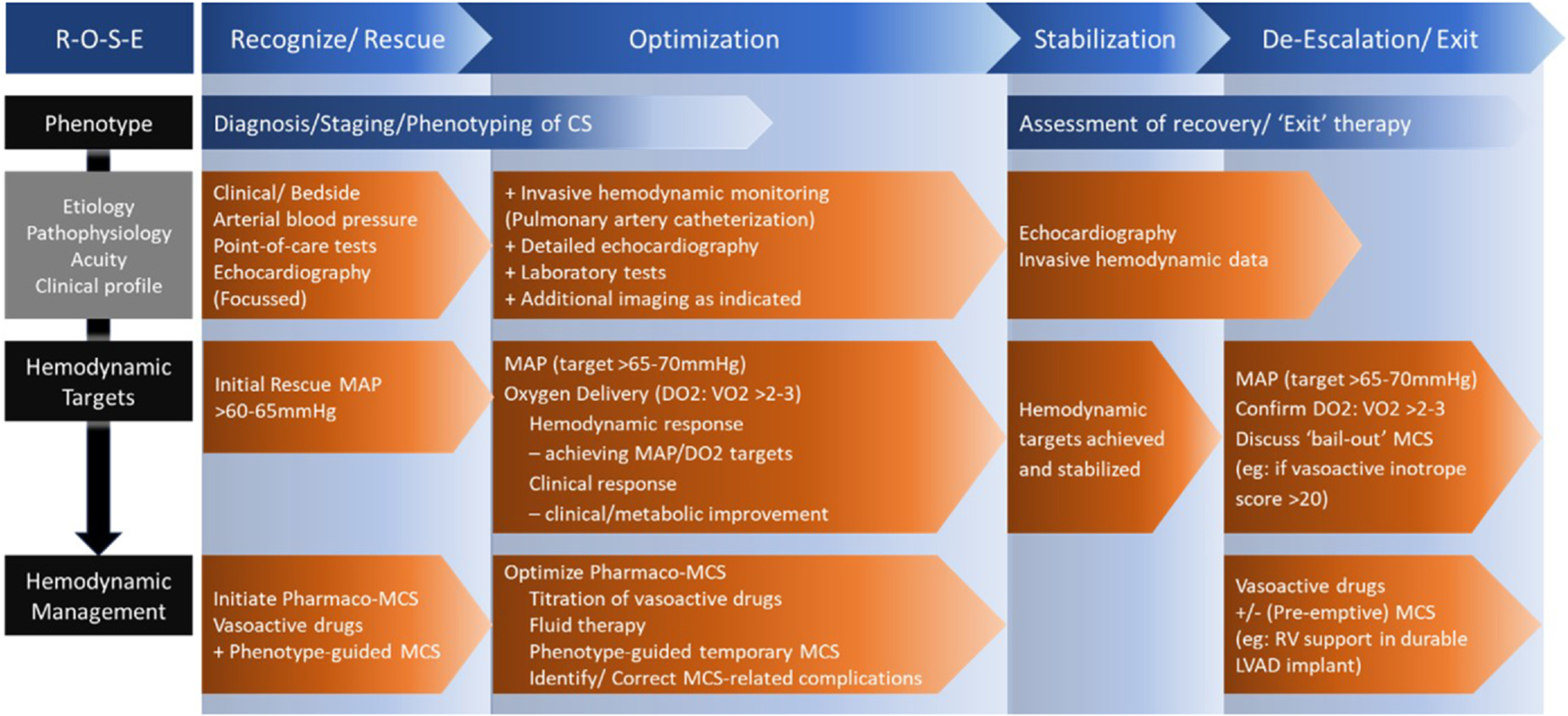
The 4 phases of CS – Recognize/Rescue, Optimization, Stabilization and de-Escalation or Exit Therapy (ROSE). During the initial phase of CS (Recognize/Rescue), assessment may be limited to clinical findings, simple hemodynamic parameters (arterial BP and heart rhythm) and point-of-care tests; and hemodynamic management and therapeutic targets are equally limited. However, successful ‘Rescue’ should be followed by a period of ‘Optimization’, a crucial phase that includes the tailoring of pharmaco-MCS therapy based on more granular hemodynamic assessment (from invasive hemodynamic monitoring) and phenotyping, correction of treatment-related complications and extra-cardiac derangements. ‘Stabilization’ is characterized by recovery of organ function, diminishing vasoactive drug requirements (invasive hemodynamic assessment could be minimized) and preparation for ‘de-Escalation and Exit’ therapy. Patients are liberated from pharmaco-MCS therapy in the event of sufficient recovery of cardiovascular function, but alternative ‘Exit’ therapy (e.g.: heart transplantation or durable LVAD) may be considered in the absence of sufficient recovery. Vasoactive drugs may be re-initiated and invasive hemodynamic assessment may need to be re-introduced for close monitoring following liberation from temporary MCS. In selected cases, de-escalating temporary MCS as staged weaning or even re-institution of temporary MCS in the event of hemodynamic deterioration after liberation may be considered. CS, cardiogenic shock; LVAD, left ventricular assist device; MCS, mechanical circulatory support.

As recovery of organ function and an ‘Exit Therapy’ are pre-requisites for survival from CS, it follows that these should be the therapeutic goals in CS, i.e.: (1) restore tissue perfusion and organ function, and (2) facilitate recovery of cardiac function, or (3) in the absence of adequate recovery, bridging to an ‘Exit Therapy’. These goals are pursued in parallel during the CS therapeutic ‘journey’. The CS ‘journey’ can be conceptualized with the additional dimension of time into 4 phases: Recognize/Rescue – Optimization – Stabilization – de-Escalation/Exit Therapy (R-O-S-E) (Figure 1). The therapeutic goals vary with the phases of care, which in turn are highly context-dependent. In some cases, the Rescue and Optimization phases may be brief, and the therapeutic goals easily achieved by introduction/titration of inotropes. In other cases, Rescue phase may necessitate deployment of veno-arterial extracorporeal membrane oxygenation (VA ECMO) as rescue therapy, followed by a protracted Optimization phase that includes hemodynamic management such as titration of vasoactive drugs, fluid therapy and escalation of temporary MCS such as the addition of temporary left ventricular assist device (LVAD) to VA ECMO (Supplementary Material).

## Phenotyping cardiogenic shock to guide treatment

**Figure 2:**
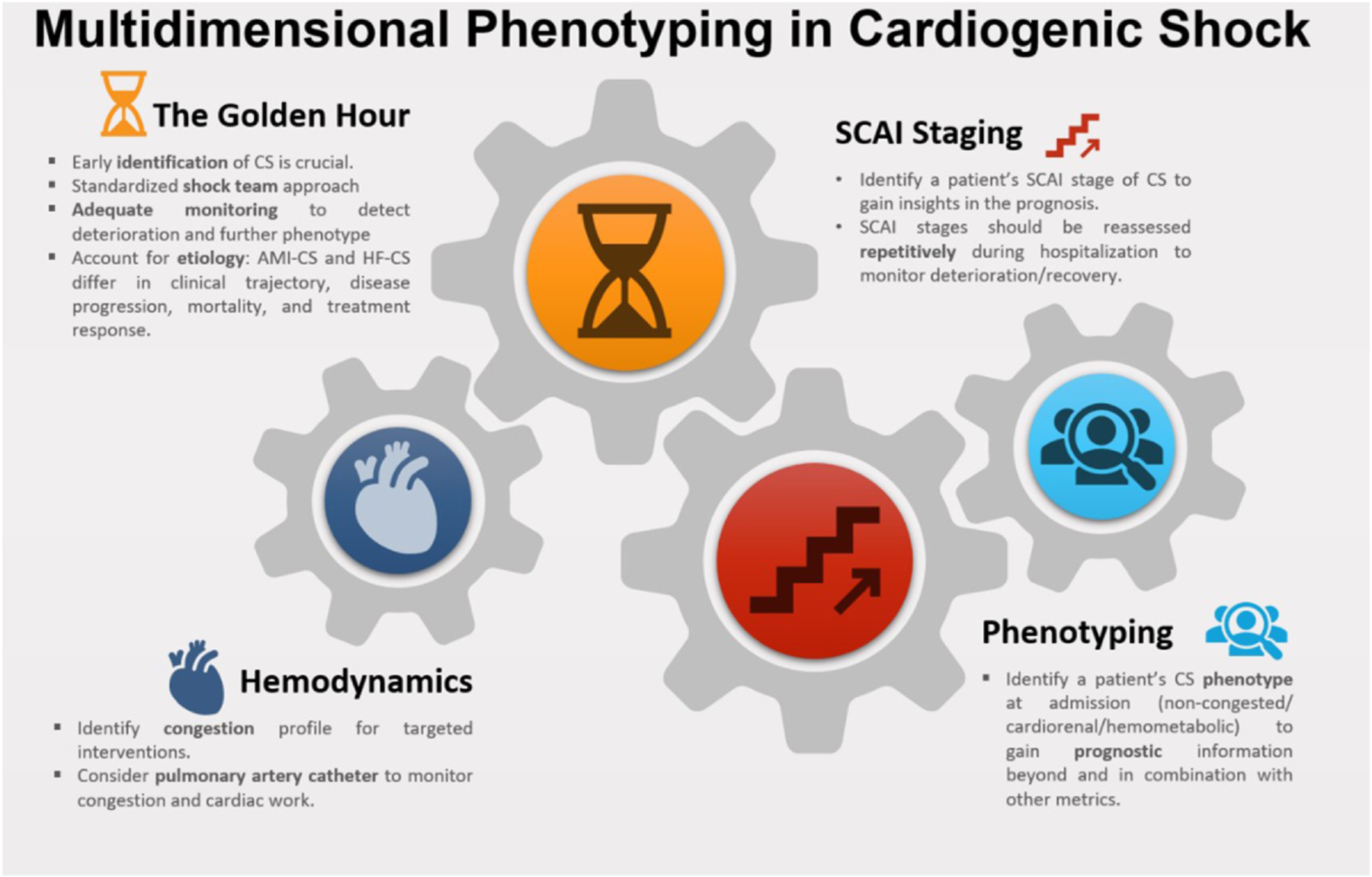
State-of-the-art phenotyping of CS patients should include a harmonization of different classification systems considering etiology, hemodynamic profiles, risk scores, SCAI staging, and machine-learning based phenotypes. While RCT-data for the emerging different CS subtypes are lacking, combining these metrics may provide carers with standardized means for risk stratification, treatment decision making and early detection of deterioration. (Design by PresentationGO.com). CS, cardiogenic shock; RCT, randomized controlled trial; SCAI, Society for Cardiovascular Angiography and Interventions.

The aims of phenotyping of CS are 2-fold: to facilitate risk stratification and tailor patient-specific management strategies (Figure 2). The premise is that a phenotype-based tailoring of pharmaco-MCS strategy improves timely delivery of appropriate intervention, thereby increasing the likelihood of response to treatment and minimizing treatment-related complications. To the clinician, phenotyping refers to the identification of a collection of observable characteristics in the patient. In machine-learning, this translates into (supervised or unsupervised) clustering algorithms that group individuals based on degrees of similarity or dissimilarity. For this review, CS phenotyping includes the characterization of:

- Underlying etiology,
- Pathophysiology,
- Severity/acuity and,
- Clinical profiles using different modalities.

Phenotyping of CS is dynamic, from rudimentary phenotyping based on limited clinical and hemodynamic data at the ‘Rescue’ phase to more granular phenotyping at the ‘Optimization’ phase with additional data from invasive hemodynamic monitoring and the benefit of information gleaned over time.

Underlying etiology: CS has been broadly divided into AMI-CS and heart failure-related cardiogenic shock (HF-CS), as these are the most common causes of CS. However, HF-CS is highly heterogenous in underlying etiology (e.g.: from familial dilated, sarcomeric and restrictive cardio-myopathies to self-limiting myocarditis), presentation (de novo vs acute-on-chronic decompensation)^3^ and cardiac phenotype. For the sake of brevity and simplicity, HF-CS henceforth refers to the dilated cardiomyopathy cardiac phenotype.

In most cases, AMI-CS presents abruptly in patients without prior history of HF.^4^ In contrast, patients with HF-CS may be encumbered by chronic sarcopenia, congestive cardio-renal-hepatic dysfunction, functional mitral/tricuspid regurgitation, pulmonary vascular disease and effects of long-term loop diuretic and neurohormonal antagonist therapy. As a result, patients with HF-CS compared to AMI-CS present with higher filling and pulmonary artery pressures, lower oxygen delivery and increased hemoglobin P50, but less severe metabolic acidosis.^5^ Pulmonary vascular disease associated with HF-CS may have implications for the management of HF-CS, not least candidacy for heart transplantation.^6^ The use of selective pulmonary vasodilators is not recommended in patients with pulmonary hypertension due to left heart disease (Group 2 pulmonary hypertension).^7^ The management of Group 1 pulmonary hypertension in the intensive care unit is beyond of the scope of this review and has been reviewed elsewhere.^8^

The treatment, clinical trajectory and outcomes in AMI-CS and HF-CS differ. Patients with HF-CS are more likely to undergo advanced therapies, including heart transplantation or durable LVAD.^9^ Some studies suggest higher short-term mortality in patients with AMI-CS compared to HF-CS.^10^ In patients with AMI-CS, the benefit of early revascularization,^11,12^ especially of the culprit lesion-only^13,14^ is supported by well-conducted RCTs that were based on sound premises and hypothesis.

Pathophysiology: Pathophysiology in the context of phenotyping involves determining the predominance of right, left, or biventricular dysfunction. Biventricular congestion has been reported in nearly 50% of patients with CS (from invasive assessment of cardiac filling pressures) and is associated with increased shock severity and higher mortality.^15^ Characterization of the pathophysiology is central to the delivery of temporary MCS. Pulmonary artery catheters (PACs) are increasingly used to characterize the pathophysiology over time to tailor pharmaco-MCS therapy. Numerous measured and derived hemodynamic parameters have been described (Table 1), but hemodynamic data from PACs can be mis-interpreted by less experienced practitioners, potentially leading to inappropriate interventions. The use of PACs should be rigorously standardized^16^ and practitioners appropriately trained, analogous to the use of other modalities such as echocardiography. Retrospective data have suggested that the use of PACs by experienced personnel is associated with improved in-hospital mortality in CS.^17–19^ The ongoing PACCs trial (ClinicalTrials.gov: NCT05485376) may provide real-world, contemporary evidence regarding the benefits of PAC in HF-CS.

**Figure 3:**
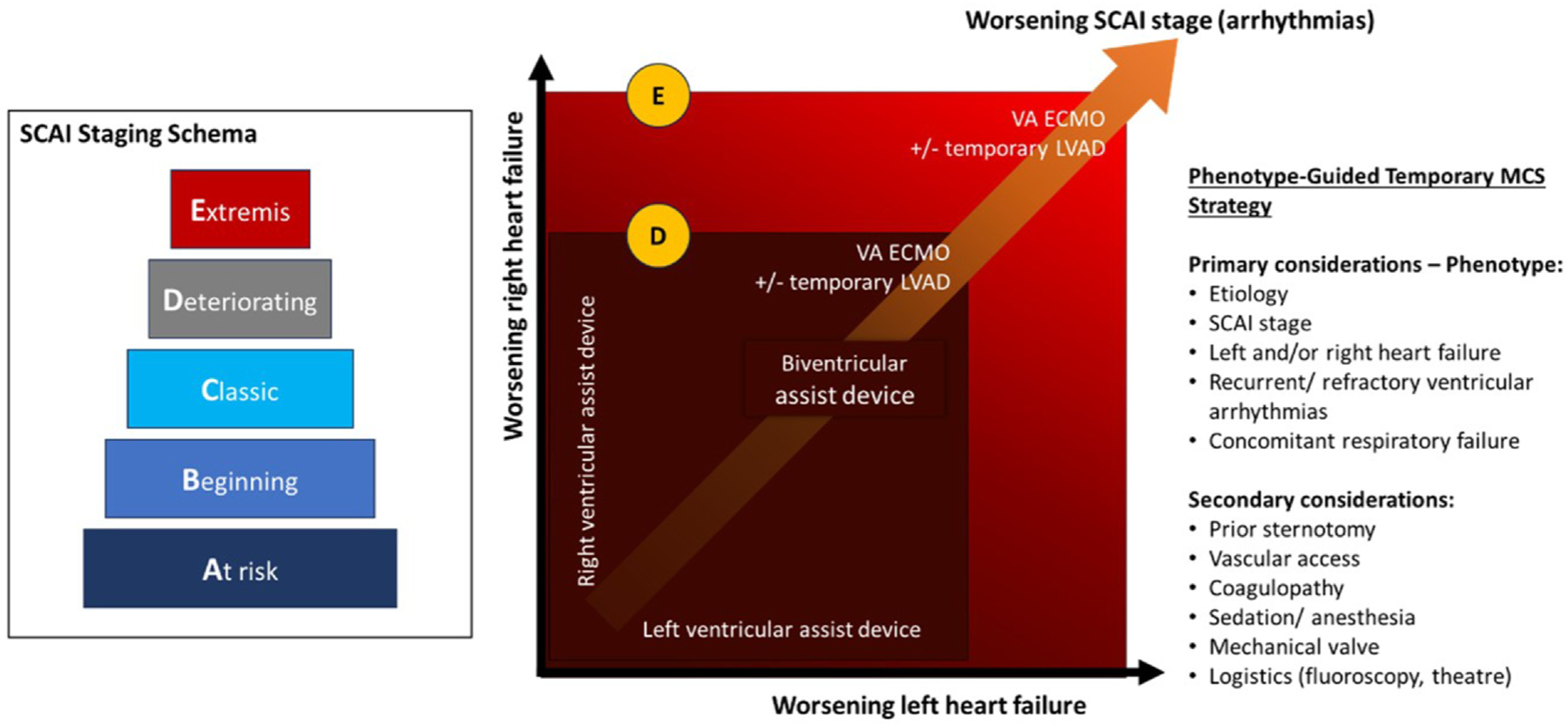
Phenotyping of CS (incorporating the SCAI staging schema and pathophysiology) should produce actionable insights to guide treatment. The premise is that phenotype-guided intervention results in timely and appropriate pharmaco-MCS therapy (“the right support for the right patient at the right time”). A temporary MCS strategy based on the SCAI staging schema and pathophysiology is presented. Temporary MCS platforms should be selected to restore tissue perfusion with the minimal morbidity and cost. CS, cardiogenic shock; MCS, mechanical circulatory support; SCAI, Society for Cardiovascular Angiography and Interventions.

Severity/Acuity: The 2019 (updated in 2022) Society for Cardiovascular Angiography and Interventions (SCAI) classification provides a schema for the assessment of CS severity.^20,21^ Retrospective and prospective studies have shown increasing risk of mortality with increasing SCAI stage.^22,23^ Applied serially, the SCAI classification can be used to assess a patient’s clinical trajectory.^24^ The SCAI schema guides the timing of intervention and the modality of temporary MCS (Figure 3). Veno-arterial ECMO is easy to deploy and highly effective in providing hemodynamic support irrespective of the pathophysiologic phenotype. This is the rationale for VA ECMO in selected patients with SCAI stage E CS.

Other clinical sub-phenotypes: Clustering algorithms using different statistical methods independently applied to retrospective CS registries have identified 3 CS sub-phenotypes with distinct clinical features and prognosis.^25,26^ Main features of these sub-phenotypes can be broadly described as: (1) “Non-congested” or “baseline” sub-phenotype with the lowest in-hospital mortality; (2) “Cardiorenal” sub-phenotype, comprised of older patients with more pre-existing co-morbidities, anemia, renal impairment and left-sided congestion; (3) “Cardiometabolic” or “hemometabolic” sub-phenotype with right-sided congestion, highest lactate levels, transaminases and in-hospital mortality. Such sub-phenotyping may improve risk stratification in combination with the SCAI classification.^27,28^

## Hemodynamic parameters as therapeutic targets in cardiogenic shock

**Figure 4:**
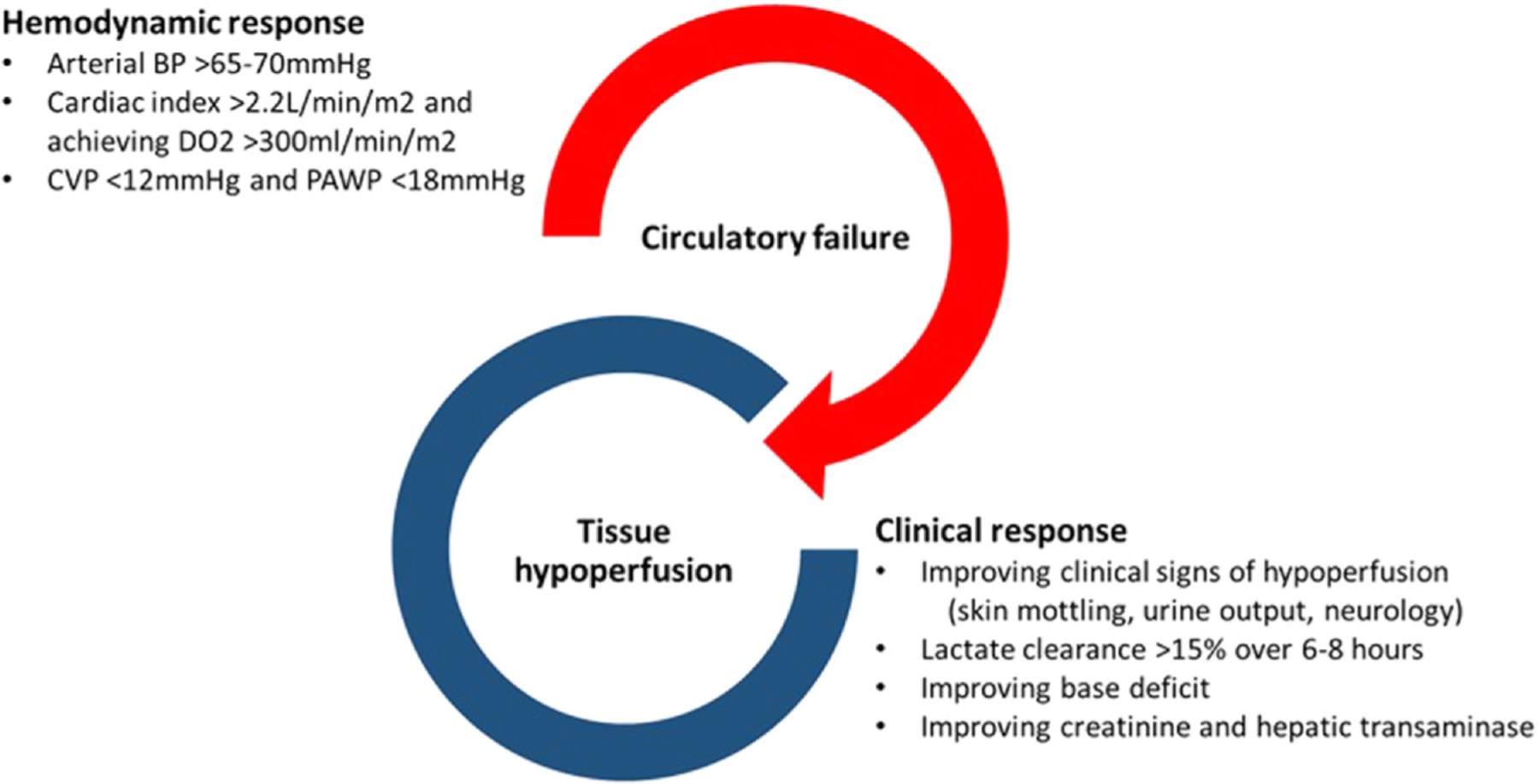
Improving hemodynamic parameters is a pre-requisite for reversing tissue hypoperfusion in CS. There is no specific treatment that can improve tissue hypoperfusion independent of hemodynamic parameters. Hemodynamic response is defined when hemodynamic targets are achieved. Hemodynamic response is not synonymous with clinical response, which is defined by improvement in markers of hypoperfusion and organ function. Hemodynamic improvement serves as an early marker of response to treatment but is not always accompanied by clinical improvement. In contrast, clinical improvement takes time to manifest (e.g.: lactate clearance is measured over 6–12 hours and improvement liver enzymes may only be evident after hours), but when present is strongly associated with improved survival. Improving survival in CS necessitates hemodynamic and clinical response to treatment and a successful Exit Therapy. CS, cardiogenic shock.

Early CS is associated with hemodynamic coherence (improvement in global hemodynamic parameters produces parallel improvement in tissue perfusion, i.e.: concordance between macro- and micro-circulation).^29^ The loss of hemodynamic coherence with prolonged periods of shock dissociates clinical response from global hemodynamic response due to microcirculatory abnormalities and cellular dysoxia. At present, tissue hypoperfusion can only be corrected by improving global hemodynamic parameters (oxygen delivery,arterial BP and reducing congestion). Based on these premises, pharmaco-MCS therapy should be instituted early in CS and target global hemodynamic parameters to correct tissue hypoperfusion. Clinical response to pharmaco-MCS therapy should not be conflated with hemodynamic response, the former is measured by the resolution of hypoperfusion, while the latter is simply the attainment of hemodynamic targets (Figure 4).

## Arterial blood pressure and vasopressors

Arterial BP measurements are ubiquitous and may be the only parameter available to guide treatment (e.g.: during the ‘Rescue’ phase). There are no established arterial BP targets in CS. Mean arterial BP of 60–65 mm Hg, extrapolated from other types of circulatory shock^30^ is a pragmatic therapeutic target during the ‘Rescue’ phase. This target is also indirectly supported by the BOX trial^31^ (no significant difference in death/disability between mean BP target of 63 mm Hg vs 77 mm Hg in patients who had been resuscitated after an out-of-hospital cardiac arrest of presumed cardiac cause).

However, hemodynamic targets are not static. Arterial BP target may need to be revised during ‘Optimization’ depending on clinical response and phenotype. A sub-study of the Milrinone as Compared with Dobutamine in the Treatment of Cardiogenic Shock trial suggested poorer outcomes in patients with 36-hour-averaged mean arterial BP < 70 mm Hg.^32^ The study by Burstein et al suggested that the sub-group of patients with HF-CS had better clinical outcomes with average mean arterial BP of > 70 mm Hg.^33^ In patients with AMI-CS and cardiac arrest, maintaining mean BP > 80 mm Hg was associated with less myocardial damage compared to mean BP target of > 65 mm Hg.^34^ It is plausible that higher BP, especially diastolic BP, improves coronary perfusion pressure (diastolic BP–end-diastolic pressure), which may be beneficial in CS complicated by elevated filling pressures. A higher mean arterial BP target of 65–70 mm Hg may be considered during ‘Optimization’, especially if clinical response is inadequate. Arterial BP should be considered in parallel with other hemodynamic parameters such as central venous pressure and cardiac index.

Norepinephrine is generally regarded as the first-line vasopressor. There is also increasing consensus on a catecholamine-sparing strategy (generally with the addition of vasopressin at 0.03–0.06 U/min)^35^ at norepinephrine doses of > 0.2 mcg/kg/min. High-dose vasopressor therapy in pursuit of higher arterial BP targets could worsen tissue perfusion, as exogenous vasoconstrictors overwhelm regional control of vascular tone, resulting in regional tissue hypoperfusion.

## Cardiac output and oxygen delivery

There is general agreement that improving global oxygen delivery (DO_2_) is the main therapeutic objective; by extension, increasing cardiac output the therapeutic target in CS. Increasing DO_2_ to 3 times that of oxygen consumption (VO_2_) in CS has been proposed,^36^ as pathological supply dependence develops below a DO_2_:VO_2_ ratio of about 2^37^ (this critical point of supply dependence may be higher in the presence of microcirculatory abnormalities).

Oxygen delivery, indexed to body surface area is the product of CI and arterial blood oxygen content:

$$\text{Indexed}\;{\text{DO}}_{\text{2}}=\text{CI}\times \left(1\cdot 36\times \text{Hb}\times \%\;\text{saturation}\right)+(0\cdot 003\times {\text{PO}}_{\text{2}})$$

**Figure 5:**
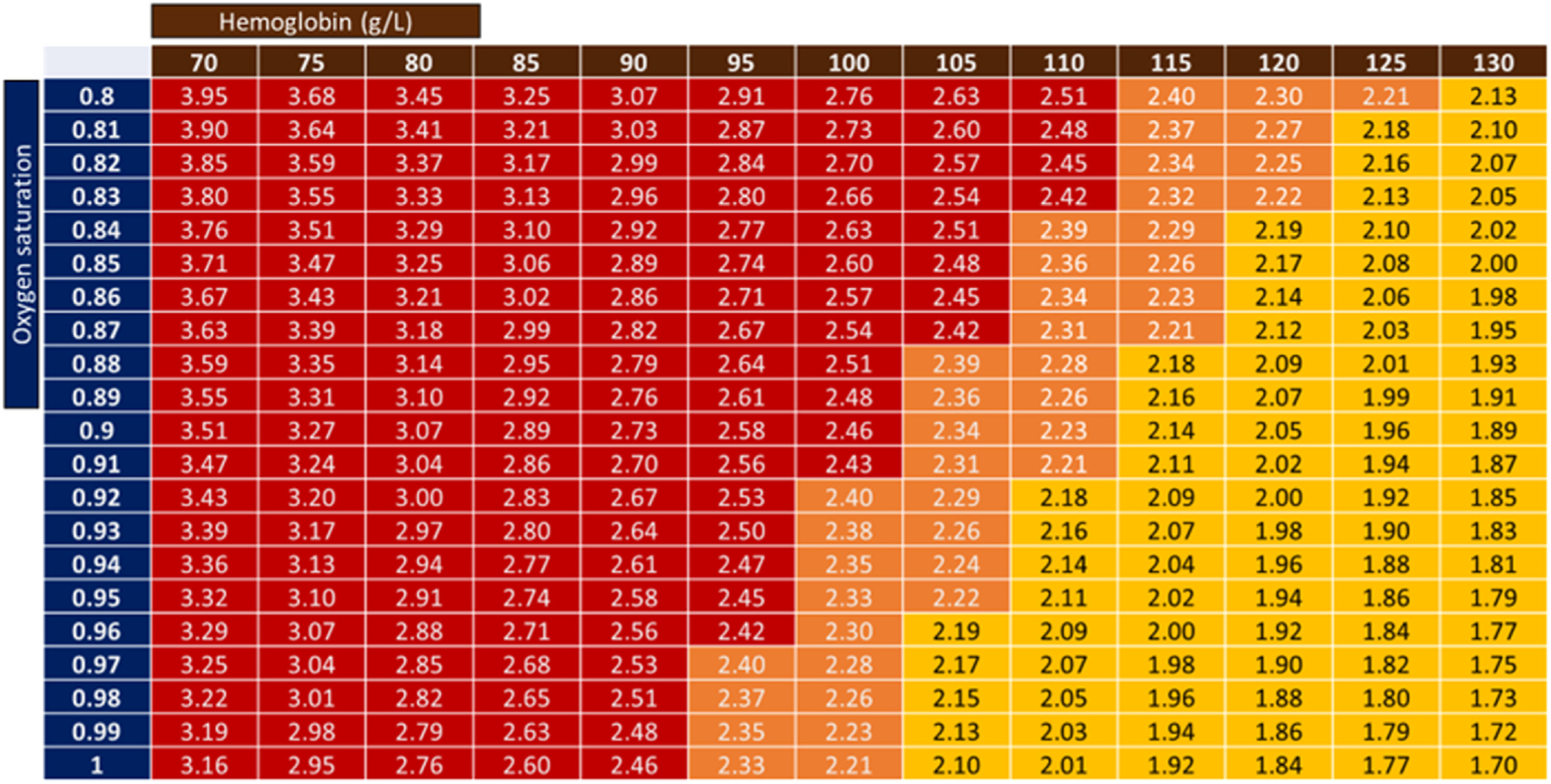
The calculated CI required to achieve a DO_2_ of 300 ml/min/m^2^ based on different levels of hemoglobin concentration and oxygen saturation. The color legend indicates the level of CI required. Lowering VO_2_ will reduce the DO_2_ requirements and the corresponding target CI. This figure does not include the intrinsic variability in cardiac output measurements – the CI target may need to be 12–15% higher to accommodate for the variability in measurements. DO_2_, oxygen delivery; VO_2_, oxygen consumption.

Resting VO_2_ from direct metabolic measurement varies from 100–150 ml/min/m^2^ in critically ill patients,^38^ which is comparable to patients with HF (100–120 ml/min/m^2^).^39^ Therefore, the critical level of DO_2_ is ≥300 ml/min/m^2^ in the critically ill patient to achieve DO_2_:VO_2_ ratio of 2–3. A higher level of DO_2_ of 450 ml/min/m^2^ may be indicated in some patients (e.g.: febrile, heavy work of breathing), although VO_2_ may also be reduced with sedation and neuromuscular blockade (8–10% reduction).^40^ Supra-normal target DO_2_ levels of 550–600 ml/min/m^2^ are now obsolete. To achieve target DO_2_ of 300 ml/min/m^2^, the target CI can be defined based on the prevailing hemoglobin concentration and oxygen saturation (Figure 5). An initial target CI of 2.2 liter/min/m^2^ is acceptable in most cases, but this target, like arterial BP may be adjusted/revised based on clinical response during ‘Optimization’.

Inotropes are used as an initial therapy for more than 90% of patients admitted to the intensive care unit with CS.^41^ Inotropes alone may produce sufficient increase in CI and DO_2_ to improve tissue perfusion in patients with SCAI stage C CS. However, patients with SCAI stages D/E CS exhibit more frequent and excessive vasodilation^42^ due to the loss of compensatory vasoconstriction.^43^ The addition of vasopressors is usually required as vasodilatation reduces the ‘gain’ (increase in BP per unit increase in output) in the systemic circulation. In many cases, vasoactive drugs, even when used in combination, may be inadequate in restoring tissue perfusion in patients with SCAI stages D/E CS^44^; or the doses required to achieve the desired hemodynamic targets result in net harm by stimulating hemodynamically significant arrhythmias, impeding cardiac recovery through increased myocardial oxygen consumption,^45^ and/or causing unwanted or excessive vasodilation/vasoconstriction and exacerbating hypoperfusion.

The few comparative RCTs of inotrope and vasopressor therapy have shown that:

- Dobutamine and milrinone have comparable effects on short-term survival,^46^
- Dopamine as vasopressor (> 10 mcg/kg/min) compared with norepinephrine was associated with increased mortality,^47^
- Epinephrine compared with norepinephrine was associated with a higher incidence of refractory CS and mortality in AMI-CS.^48,49^

Inotropes commonly used in CS include dobutamine, phosphodiesterase inhibitors (milrinone/enoximone), dopamine and epinephrine. The choice of inotrope varies between centers, based on: (1) the results of RCTs; (2) putative benefit in specific patient populations (e.g.: HF-CS); (3) heart rate and perceived risk of tachyarrhythmias; (4) institutional experience/preference, and (5) access to the vasoactive drug in specific healthcare systems (e.g.: levosimendan). In general, dobutamine is commonly used as the first-line agent, but phosphodiesterase inhibitors may be the preferred agent in HF-CS, especially in patients on beta-blocker therapy. Dopamine is used in some case (at lower doses (< 10 mcg/kg/min) if heart rate is relatively low. Epinephrine is generally regarded as a second (and even third)-line inotrope or vasopressor.^50^

Vasodilators such as sodium nitroprusside (SNP) is rarely used in CS due to the common presentation with hypotension. However, SNP may have a role in selected patients with HF-CS. In patients with left ventricular dilatation, functional mitral regurgitation, low ejection fraction and less severe/acute CS (SCAI stages B and C), SNP has been shown to simultaneously increase stroke volume and lower pulmonary artery wedge pressure (PAWP), without inducing symptomatic hypotension. Indeed, in many cases, the highest stroke volume was achieved at the lowest PAWP.^51^ The improvement in loading conditions with SNP is also associated with improvement in functional mitral regurgitation.^52^ However, SNP is more likely to induce hypotension and reduce stroke volume in patients with relatively normal chamber volumes and preserved left ventricular ejection fraction,^53^ and should be avoided.

**Figure 6:**
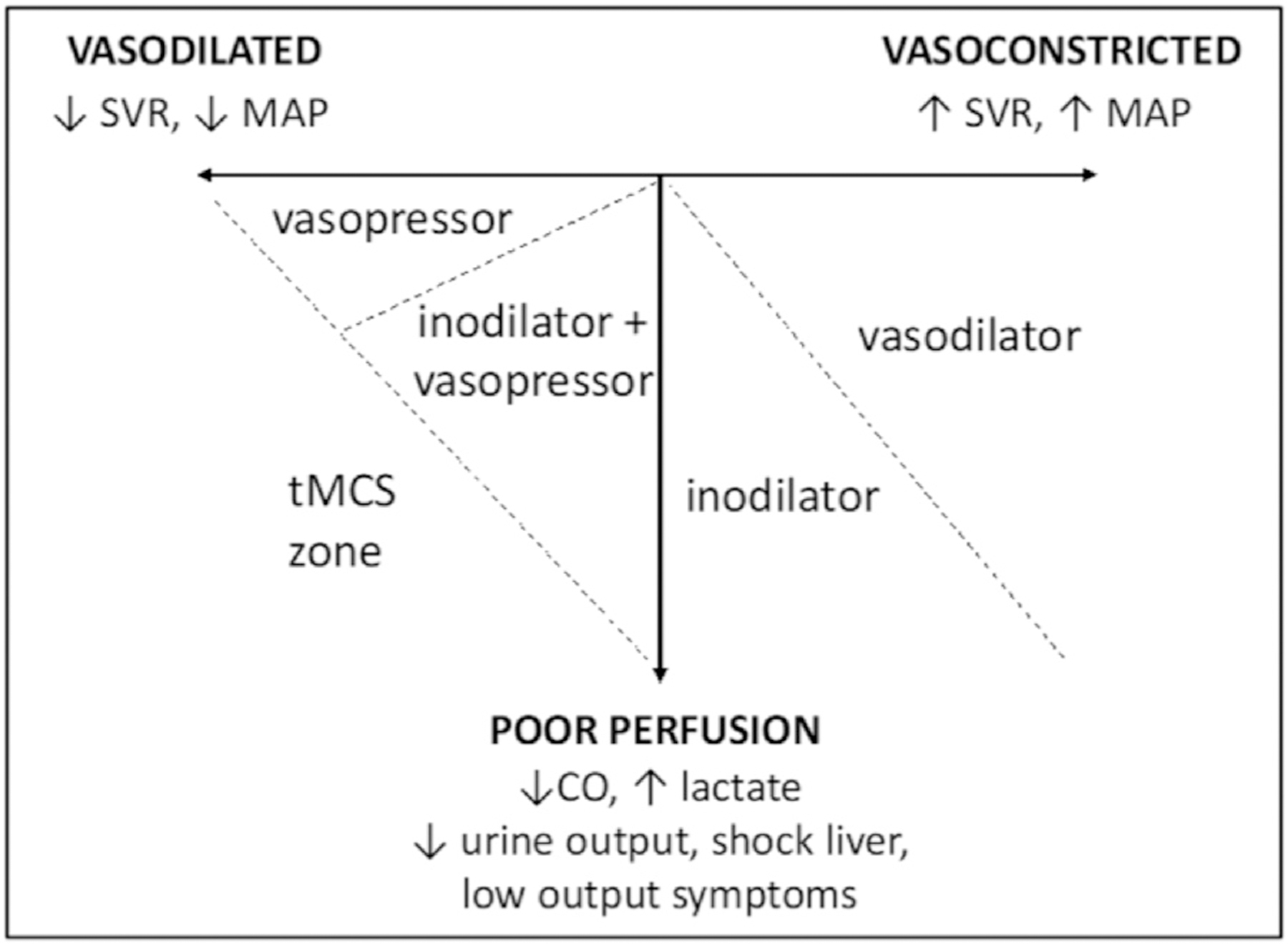
Hemodynamic-guided optimization of vasoactive medications and temporary MCS in CS. Patients with modest reductions in cardiac output may benefit from inotropic support alone to improve tissue perfusion. Vasopressors or vasodilators may be utilized alone or in conjunction with inotropes (or by selecting inotropes with favorable vasoconstriction or vasodilating effects) depending on the degree of pathophysiologic vasoconstriction or vasodilation present. Patients with severe reductions in both cardiac output and systemic vascular resistance may require escalation to temporary MCS to adequately restore tissue perfusion while maintaining acceptable perfusion pressure. CS, cardiogenic shock; MCS, mechanical circulatory support.

Temporary MCS devices are indicated for additional hemodynamic support, oxygen delivery and vasoactive drug-sparing effects ( Figure 6) and should be considered in selected patients based on pre-existing co-morbidities, likelihood of recovery and potential candidacy for ‘Exit’ therapy. Temporary MCS should be matched to the patient phenotype. In cases of phenotypic uncertainty, invasive hemodynamic monitoring may aid hemodynamic phenotyping and guide the choice of temporary MCS modality. Centers will have variable thresholds in offering temporary MCS based on institutional practices and experience. Several factors were identified through a study utilizing the RAND Appropriateness Panel approach as inappropriate for temporary MCS^54^ (Table 2). Other contra-indications include significant dementia and frailty, irreversible organ failure (e.g.: liver cirrhosis) and metastatic malignancy.

## Optimization of pharmaco-mechanical circulatory support therapy

Hemodynamic management during the ‘Optimization’ phase includes:

- Titration of complementary pharmaco-MCS therapy to improve DO_2_:VO_2_ balance and perfusion pressure to reverse tissue hypoperfusion.
- Minimize and correct adverse effects of pharmaco-MCS.

Hemodynamic and clinical response to pharmaco-MCS therapy should be continuously assessed during ‘Optimization’ – the Boydian loop of “Observe-Orient-Decide-Act” (Supplementary Material). For example, high norepinephrine requirements prior to support (indicative of relative vasodilatation) are associated with poor hemodynamic response to microaxial LVAD and need for escalation.^55^ In contrast, improvement in hemodynamic status following initiation of temporary MCS may allow reduction in the (high) doses of vasoactive drugs from the ‘Rescue’ phase to minimize adverse effects and reduce myocardial oxygen consumption towards the goal of cardiac recovery. Similarly, temporary MCS flow should be “dosed” to balance DO_2_ and unloading goals with the risk of hemolysis and coagulopathy, driven by flow-dependent blood shear stress.^56^

All temporary MCS devices increase the risk of bleeding, limb ischemia, vascular damage, hemolysis, and infection to varying degrees. In 1 contemporary multi-center study, device-related complications occurred in ∼50% patients treated with VA ECMO and were associated with a worse prognosis.^57^ These risks may be offset by more rapid and complete restoration of tissue perfusion. Temporary LVAD (catheter-mounted microaxial LVAD) are also associated with significant risks of major complications including limb ischemia (10%) and life-threatening bleeding (8–10%).^58^ The combination of VA ECMO and catheter-mounted microaxial LVAD may be associated with improved short-term survival but is associated with significant morbidity including hemolysis (∼34%), severe bleeding (∼38%) and limb ischemia requiring intervention (∼22%).^59^ Many of these complications may be mitigated by a pro-active strategy to identify and prompt correction of treatment-related complications.

The potential of cardiac recovery should also be pursued, especially in cases where recovery is possible (e.g.: myocarditis) and/or recovery is the only ‘Exit’ from CS. Improving loading conditions, e.g., unloading of the left and/or right ventricle, is a vehicle to facilitate cardiac recovery. VA ECMO increases left ventricular afterload and may require pharmacologic or mechanical unloading to mitigate ventricular wall stress and pulmonary edema.^60^ Some studies suggest better survival with early (< 2 hours) addition of microaxial LVAD to unload the left ventricle during VA ECMO support, at the cost of higher incidence of complications.^61^ A full discussion on the management of temporary MCS is beyond the scope of this review.

## Perfusion and organ function as markers of clinical response

Improving hemodynamic parameters are necessary but insufficient for clinical response (defined as improvements in markers of hypoperfusion and organ function) ( Table 3). Measurement of blood lactate is now routine in circulatory shock. Classically, hyperlactatemia in CS is attributed to global or regional reduction in oxygen delivery below the critical point of supply-dependence. Reversing the state of supply dependence corrects hyperlactatemia. Thus, reduction in blood lactate over time (so-called lactate clearance) is a good marker of adequacy of tissue perfusion.^62^ Median lactate clearance at 6–8 hours was 21.9% (IQR 14.6%−42.1%) in survivors and 0.6% (IQR −3.7% to 14.6%) in non-survivors.^63^ Poor lactate clearance may be indicative of: (1) failure to achieve hemodynamic targets or inadequate hemodynamic targets; (2) persistent or irrecoverable organ ischemia/infarction; (3) loss of hemodynamic coherence due to microcirculatory dysfunction and/or cytopathic dysoxia; (4) (adrenergic) accelerated aerobic lactate production.

Clinical markers of perfusion (e.g., regression of skin mottling) and clinical or laboratory markers of organ function (e.g., urine output, creatinine and transaminase) provide specific but delayed measures of response to therapy. Direct imaging-based assessment of the microcirculation have been developed to provide rapid, non-invasive, bedside assessment of the micro-circulation, but a multi-center randomized trial that incorporated serial assessment of the sublingual microcirculation (side-stream-dark field video microscope) to guide treatment in circulatory shock (including CS) failed to improve clinical outcomes, despite significantly more changes in therapeutic interventions.^64^

## Bridging to ‘Exit’ therapy

Most would consider cardiac recovery, heart transplantation or durable LVAD as acceptable ‘Exit Therapy’ from CS. ‘Recovery’ is poorly defined probably because it is not a simple binary phenomenon, but a continuum from short-term ‘hemodynamic recovery’ (improvement in cardiac output and filling pressures) sufficient for liberation from temporary MCS, often accompanied by partial or complete ‘cardiac recovery’ (normalization of systolic and diastolic chamber volume and geometry) to long-term ‘prognostic recovery’ (return to expected survival). The pattern of recovery varies with the underlying etiology. For example, liberation from temporary MCS may be interpreted as recovery and an acceptable endpoint for RCTs in patients with HF-CS, but unlikely to be an acceptable ‘recovery’ for the patient if median survival is only 2–3 years.^65^ In contrast, survival from acute fulminant myocarditis is often accompanied by complete ‘cardiac’ and ‘prognostic’ recovery.^66^ Registry data suggest that (hemodynamic) recovery occurs in < 50% of patient with CS, especially in SCAI stages D and E.^67^

Extrapolating from data in patients with HF supported by durable LVAD (recovery defined as explant of LVAD), characteristics associated with higher likelihood of myocardial recovery include peripartum cardiomyopathy and short duration of HF (< 1 year). In contrast, ischemic cardiomyopathy, higher left ventricular diameter and lower ejection fraction (< 20%) on LVAD support were associated with lower likelihood of myocardial recovery.^68^ Like durable LVAD, temporary LVAD may bridge patients to candidacy and transplantation by reversing combined pre- and post-capillary pulmonary hypertension associated with left heart disease.^69^

In AMI-CS, the degree of circulatory embarrassment is related to infarct size, mechanical or arrhythmic complications, phenomenon of stunning and concomitant distributive shock or vasodilatation (often associated with systemic inflammatory response). Early improvement (mostly within 72 hours) in cardiac function is a common feature of cardiac stunning.^70^ Similarly, systemic inflammatory response may resolve over several days. However, larger infarct sizes may be associated with lower likelihood of recovery. Autopsy studies showed that AMI-CS related to left ventricular failure is generally associated with a loss of > 40% of left ventricular myocardium.^71^ Pathological Q waves,^72^ microvascular obstruction,^73^ no-reflow phenomenon^74^ following revascularization are associated with larger infarct sizes and higher risk of HF, arrhythmias and mortality, by extension lower likelihood of recovery.

In the absence of recovery, selected patients may undergo heart transplantation or durable LVAD as alternative ‘Exit Therapies’. Individual centers have developed strategies to bridge patients with CS to an ‘Exit Therapy’, based on local expertise and access to devices.^75^ In some series, the outcomes of temporary MCS bridging to heart transplantation^76^ and durable LVAD (1-year survival 53%)^77^ are inferior to patients without MCS bridging. However, meticulous ‘Stabilization’ prior to ‘Exit Therapy’ and anticipatory hemodynamic management following ‘Exit Therapy’ (e.g.: pre-emptive right ventricular support in durable LVAD therapy due to the higher risk of right heart failure associated with MCS bridging^78^ may offer a chance for delayed recovery and improve outcomes of MCS bridging to ‘Exit Therapy’.^79^ Discharge on palliative inotrope therapy is a poor outcome from CS (generally not regarded as a successful ‘Exit Therapy’), with median survival of several months.^80^

Temporary MCS devices, primarily as supportive therapies cannot improve survival in CS in the absence of sufficient recovery of native cardiovascular function or an ‘Exit’ (a definitive interventional/surgical treatment, heart transplantation or durable LVAD), no more than mechanical ventilation can improve survival in patients with irreversible lung failure (Table 4). This is the central premise for pharmaco-MCS therapy. Indeed, RCTs of temporary MCS devices such as intra-aortic balloon pump,^81,82^ Impella 2.5/CP^83–85^ or VA ECMO,^86–90^ built on incomplete premises have not been shown to improve survival in patients with CS, and may even increase morbidity (bleeding and vascular complications).^91^ The ‘Exit Therapy’ should be considered prior to and at all phases of CS management.

## Future directions

The future of CS management is dependent on the clinicians’ approach to patient care, trialists’ approach to RCTs and the wider societal approach to CS. Firstly, clinicians must move away from a primarily device-centric to a patient-centric approach – i.e,: tailoring pharmaco-MCS therapy to the patient instead of fitting the patient to the treatment. ‘Big data’ should be leveraged to improve the phenotyping of CS, using clustering algorithms from machine-learning to define clinical phenotypes to guide pharmaco-MCS therapy. Machine-learning models from vital signs and laboratory data may expedite the recognition of CS in patients with AMI and acute heart failure.^92^

Secondly, RCTs are experiments and are constrained by the premises that underpin their hypotheses – RCTs based on hypotheses deducted from vague or incomplete premises could be internally valid, but their results clinically ambiguous (Table 4). A dispassionate appraisal of the premises of RCTs is needed to strengthen future RCTs. The premises for future RCTs must consider hemodynamic management through the 4 phases of CS from initial Rescue to ‘Exit Therapy’. There are lessons from RCTs of acute heart failure that we will do well to heed – CS is not a 48-hour illness that can be treated with a single intervention.^93^

Thirdly, we should draw on the experience of colleagues in the field of sepsis. Almost 20 years ago, with the aim of improving the management of sepsis, an international group of clinicians developed a 6-point plan – so-called Barcelona Declaration. The Barcelona Declaration described the problems associated with sepsis, outlined a statement of intent, which served as a call to action to increase awareness of sepsis among health care professionals, governments, the public and funding bodies (Supplementary Material). Arguably, the Surviving Sepsis Campaigns that emerged from the Barcelona Declaration have been the driver behind the improvements in the global landscape of sepsis care. A similar call to action would advance the field of CS management.

## Conclusion

Hemodynamic management is the central pillar in the management of CS but has yet to take center stage in RCTs. It is hoped that the framework and the premises outlined in this State-of-the-Art will generate scientific discourse. Such discourse is necessary for progress, and on the subject of hemodynamic management of CS, is long overdue.

## Supplementary Material

Supp Material 1

Supp Material 2

Supp Material 3

Supp Material 4

## Figures and Tables

**Table 1:** Indications and Parameters from Pulmonary Artery Catheterization

Indication	Parameters
Titration of vasoactive drugs in cardiogenic shock	Cardiac index, SV, CPOi, SVR
Limited response to initial therapy and uncertain hemodynamic status:	Assessment includes measured and all derived parameters
Pulmonary vascular disease	CVP: PAWP, PAPI, RVSW, PPP
Right heart failure	TPG, PVR, DPG, PA capacitance
Mixed shock/relative vasodilatation	SVR, DSI
Optimization of diuretics and vasodilators to facilitate weaning from inotropes	CVP, PAWP, CVP: PAWP ratio
Assessment of patients for heart transplantation or left ventricular assist device	PA pressure, TPG, PVR and DPG
Assessment of intracardiac shunt	Depending on location of shunt Right atrial and PA oxygen content
*Derived parameters and calculation*	*Comments/cutoff*
Transpulmonary gradient (TPG)	Abnormal > 12–15 mm Hg
= Mean PA pressure – PAWP	Usually > 15 mm Hg in pressure-overloaded right heart failure
Pulmonary vascular resistance (PVR)	Abnormal ≥ 2WU
= Transpulmonary gradient/ cardiac output	But usually > 5WU in pressure-overloaded right heart failure
Diastolic pressure gradient (DPG)	Abnormal if > 5–7 mm Hg, suggests pulmonary vascular disease
= PA diastolic pressure – PAWP	But usually > 7 mm Hg in pressure-overloaded right heart failure
Pulmonary artery capacitance	Related to PVR
= Stroke volume/ PA pulse pressure	Lower capacitance associated with poorer outcomes < 0.81 ml/mm Hg in pulmonary arterial hypertension^94^ < 2 ml/mm Hg in heart failure^95^
Left/right filling pressures	Higher ratio associated with poorer outcomes
= CVP/PAWP	> 0.63 (post-LVAD)^96^ > 0.86 (acute MI)^97^
Right ventricular stroke work (RVSW)	Varies with PVR, lower levels associated with poorer outcomes
= (mean PA – CVP) x SV x 0.0136	< 15 (post-LVAD)^98^ < 10 (acute MI)
Pulmonary artery pulsatility index (PAPI)	Lower PAPI associated with worse prognosis, but cutoff varies with PVR^99^
= PA pulse pressure/CVP	< 1.85–3.3 (post-LVAD) < 1.0 in primary RV dysfunction without pulmonary hypertension
Proportional pulmonary pulse pressure (PPP)	Lower in right heart failure
= PA pulse pressure/mean PA pressure	> 0.60 post-VA ECMO associated with better hemodynamic response
Cardiac power output index (CPOi)	Lower CPOi associated with worse outcomes
= cardiac index x (MAP-CVP)/451	Performs better with the inclusion of CVP, especially if CVP > 8 mm Hg^100^ In CS^101^ or Impella support,^102^ cutoffs > 0.28–0.30 W/m^2^
Aortic pulsatility index (API)	Variable cutoffs, ≥1.45^103^ to ≥2.9^104^
= (SBP-DBP)/PAWP	Lower API associated with worse prognosis, mostly in patients with heart failure Limited data in CS
Left ventricular stroke work (LVSW)	Limited data in CS
= (MAP-PAWP) x SV x 0.0136	
Systemic vascular resistance (SVR)	Normal 800–1200 dyne/s/cm^−5^
= (MAP-CVP)/cardiac output	A summation of arterial, arteriolar, microvascular and venous resistance.
Or cardiac index to derive systemic vascular resistance index	
Diastolic shock index (DSI)	A measure of relative vasodilatation
= Heart rate/diastolic BP	Higher DSI associated with poorer outcomes in septic shock^105^ DSI > 2.0 in septic shock No data in CS

CS, cardiogenic shock; CVP, central venous pressure; DBP: diastolic blood pressure; LVAD, left ventricular assist device; MAP, mean arterial pressure; MI, myocardial infarction; PA, pulmonary artery; PAWP, pulmonary artery wedge pressure; SBP: systolic blood pressure; SV, stroke volume; VA ECMO, veno-arterial extracorporeal membrane oxygenation.

**Table 2:** Appropriateness of Temporary MCS for CS

Condition	Appropriateness
Clinical/imaging evidence of hypoxic brain injury	Inappropriate
Active/ uncontrolled bleeding	Inappropriate
Prohibitive vascular access	Inappropriate
Age > 80 years	Inappropriate
Shock team consensus of futility	Inappropriate
Lactate > 8 mmol/l	Uncertain
Cardiopulmonary resuscitation > 30 minutes before return of spontaneous circulation	Uncertain
Ineligibility for advanced heart failure therapies	Uncertain

CS, cardiogenic shock; MCS, mechanical circulatory support.

**Table 3:** Markers of Hypoperfusion

Parameter	Comments
*Clinical assessment*	
Cold, clammy skin	Used in clinical trials, but subjective and poorly defined. Not specific to shock states.
Capillary refill time (CRT)	In cardiogenic shock, a CRT of > 3 seconds was associated with mechanical circulatory support and 90-day mortality.^106^ Poor correlation with macrocirculatory parameters, such as blood pressure and cardiac index. Associated with reduction in lactate level. Reflects microcirculatory abnormalities. Affected by skin temperature (longer in hypothermia).
Skin mottling	A 6-degree scale to quantify skin mottling: 0 = no mottling; 1 = small mottling area (coin size) at the center of the knee; 2 = mottling area that does not exceed the superior edge of the knee cap; 3 = mottling area that does not exceed the middle thigh; 4 = mottling area that does not go beyond the fold of the groin; 5 = extremely severe mottling area that goes beyond the fold of the groin. Higher grade associated with mortality in critically ill patients,^107^ higher mortality in grade ≥ 2.^108^ Limited data in cardiogenic shock. Poor correlation with macrocirculatory parameters, reflects microcirculatory abnormalities.
New altered mental state	Not specific to shock. May be affected by medications (eg: benzodiazepines and opiates) and intra-cranial pathology. Associated with higher mortality if altered mental state is related to cardiogenic shock.^109^
Oliguria	Urine output < 0.5 ml/kg/hour. Indicative of renal impairment, not specific for cardiogenic shock. Delayed marker. Requires catheterization for accurate quantification.
*Rapid point-of-care measurement*	
Blood lactate	Levels of > 2 mmol/l are generally considered abnormal and persistent hyperlactatemia is consistent with CS. Higher level associated with higher mortality. Rapid reduction in lactate associated with better outcomes (termed ‘‘lactate clearance’’). Poor lactate clearance despite improvement in hemodynamic parameters may indicate organ ischemia/infarction, microcirculatory dysfunction and/or cellular dysoxia (loss of hemodynamic coherence). Not specific to hypoperfusion. Other causes include aerobic glycolysis (eg: epinephrine) and drugs (eg: metformin).
Base excess	Deficits (negative) in base excess indicative of renal dysfunction. Renal dysfunction not specific to cardiogenic shock. May be affected by medications (eg: furosemide).
Near-infrared spectroscopy (NIRS)	Based on differential absorption spectrums of oxygenated and deoxygenated hemoglobin, in small, mostly venous vessels (< 1 mm). Tissue hemoglobin oxygen saturation (StO2) can be calculated, which represents the local balance between oxygen delivery and consumption. NIRS-derived StO_2_ can predict mortality in circulatory shock, but the impact of NIRS monitoring on outcomes is uncertain. Standardization of methodology and clinical randomized trials are needed before wider clinical use
*Invasive bedside assessment*	
Mixed venous oxygen saturation (SvO_2_)	Typically measured from pulmonary artery The relationship between central and mixed venous oxygen saturation. Lower at higher levels of oxygen extraction, due to inadequate oxygen delivery and/or increased oxygen consumption. There is not a single SvO_2_ cut-off to define hypoperfusion in CS, as low SvO_2_ can be tolerated in some patients with advanced HF without CS.^110^ Central and SvO_2_ in CS due to AMI are typically < 50–55% and lower in decompensated chronic HF.^111–113^ May paradoxically be normal (> 70–75%) or supranormal in severe CS due to microcirculatory and/or cellular dysoxia. Serial assessment may identify deteriorating CS.
Venous-arterial gradient in the partial pressure CO_2_ (ΔPvaCO_2_)	Small gradient normally (< 6 mm Hg or 0.8 kPa). Related to low flow and not specific for hypoperfusion. Minor measurement error will have significant impact. Requires central venous cannulation.
Ratio of ΔPvaCO_2_ to arterial-venous oxygen content (ΔPvaCO_2_: ΔCavO_2_ ratio)	Ratio of > 1.4 mm Hg.dl/mlO_2_ Indicative of anaerobic metabolism. Cumbersome calculation. Little data in cardiogenic shock. Requires central venous/ pulmonary artery catheterization.
*Organ dysfunction*	
Creatinine	Not immediately available and non-linear relationship with glomerular filtration rate. Usually delayed/ late marker.
Liver transaminase and bilirubin	Kinetics of transaminase more rapid than bilirubin (bilirubin may lag behind by 48 hours).
Platelet count	Thrombocytopenia is common, especially with mechanical circulatory support, reflects severity of shock.

AMI, acute myocardial infarction; CRT, capillary refill time; CS, cardiogenic shock; HF, heart failure; NIRS, near-infrared spectroscopy; ΔPvaCO_2_, venous-arterial gradient in the partial pressure CO_2_; ΔPvaCO_2_, ΔCavO_2_ ratio: ratio of ΔPvaCO_2_ to arterial-venous oxygen content; SvO_2_, mixed venous oxygen saturation.

**Table 4:** The Premises for Deductive Hypotheses in Clinical Trials and Clinical Practice

Revascularization in AMI-CS	
Premises	Deductive hypothesis
Better cardiac function increases the likelihood of survival from AMI-CS. Early revascularization in AMI reduces infarct size and minimizes the degree of cardiac dysfunction.	Early revascularization improves survival in AMI-CS.
Comments:	
This hypothesis has been proven. Early revascularization in AMI-CS is now well-established. In addition, revascularization of the culprit vessel only is preferable to multi-vessel or full revascularization.	
*Hemodynamic management in CS*	
Premises	Deductive hypotheses
Low DO_2_:VO_2_ due to cardiac output limitation, low perfusion pressure and pathological regional vasoconstriction are dominant causes of tissue hypoperfusion and organ dysfunction in CS. Early CS is characterized by hemodynamic coherence and improving global DO_2_ improves tissue hypoperfusion. Tissue hypoperfusion, at present can only be corrected by improving global hemodynamic parameters. Hemodynamic and clinical responses to pharmacological treatment and/or MCS devices are related to clustering of observable patient characteristics (phenotype). A pharmaco-MCS strategy tailored to the patient phenotype improves clinical response and minimizes treatment-related complications.	Early targeting of hemodynamic parameters such as arterial blood pressure, filling pressures and global DO_2 improves tissue hypoperfusion and organ dysfunction in CS._ A phenotype-guided hemodynamic management strategy is superior to ‘generic’ hemodynamic management strategy.
Comments:	
Hemodynamic management of CS has not been examined in randomized trials. In other forms of circulatory shock, most notably sepsis, early goal-directed therapy has been extensively investigated in randomized trials and adopted as standard of care in many institutions. Calls for greater use of pulmonary artery catheterization are founded on the premise that the hemodynamic insights from pulmonary artery catheterization improves pathophysiologic phenotyping and tailoring of therapy.	
*Vasoactive drugs in CS*	
Premises	Deductive hypotheses
Selective use of vasoactive drugs with complementary pharmacodynamic effects can produce more favorable hemodynamic responses and minimize dose-related toxicity. Invasive hemodynamic monitoring can guide the selective use of complementary vasoactive drugs at the lowest dose necessary to improve hemodynamic response and minimize drug-related adverse effects.	A strategy of selective vasoactive drug therapy guided by invasive hemodynamic monitoring improves hemodynamic response.
Comments:	
RCTs have tested vasoactive drugs in isolation but these drugs are often used in combination based on mechanism of action, and frequently with invasive hemodynamic monitoring. Emerging concepts such as “broad-spectrum vasopressor” and “catecholamine-sparring” strategies are based on this premise.	
*Temporary MCS in CS*	
Premises	Deductive hypotheses
The severity of hemodynamic compromise and tissue hypoperfusion are dominant predictors of early mortality in CS. Early temporary MCS (in the setting of hemodynamic coherence) reduces catecholamine requirements, improves DO_2_ and tissue hypoperfusion. Survival from CS is dependent on: The absence of severe, irreversible organ dysfunction (eg: hypoxic brain injury), and The presence of an ‘Exit’ therapy. Temporary MCS cannot improve survival in CS without adequate cardiac recovery in the absence of an alternative ‘Exit’ therapy.	Early temporary MCS, compared to delayed temporary MCS improves hemodynamic status and hypoperfusion in patients with CS. Temporary MCS compared to medical therapy improves survival in patients with severe CS in the presence of an ‘Exit’ therapy.
Comments:	
‘Exit’ therapy refers to recovery of cardiovascular function, heart transplantation or durable LVAD, or other medical/ surgical/ interventional procedures that can result in adequate improvement in cardiovascular function that is commensurate with long-term survival. The premise that temporary MCS would improve survival in the absence of an ‘Exit’ therapy is not plausible, unless temporary MCS enhances the probability of cardiac recovery.	

AMI, acute myocardial infarction; CS, cardiogenic shock; DO_2_, oxygen delivery; LVAD, left ventricular assist device; MCS, mechanical circulatory support; RCTs, randomized controlled trials; VO_2_, oxygen consumption.
