# Supplementary material for: Hemodynamic management of cardiogenic shock in the intensive care unit
Source: J Heart Lung Transplant. Author manuscript; Available in PMC 2025 Jul 1. (PMC11148863; doi:10.1016/j.healun.2024.03.009)
Supplement: Supp Material 1 [file NIHMS1990686-supplement-Supp_Material_1.docx]

| **Results** | No change in ΔCI (p=0.43); but increased refractory CS (37% with Epinephrine vs. 7% with Norepinephrine; p=0.0008) | Median hs-cTnT of 1.14 ug.72 h/L in higher MAP target vs. median hs-cTnT of 1.56 ug.72 h/L in lower MAP target | Median LVEF of 50 (IQR: 44-59)% in ECLS vs median LVEF of 50.8 (IQR: 47-61)% in Control. No difference in 1 year mortality. | RR: 0.90 (0.69-1.19); p=0.47 | ΔCO: 61 ±22 L vs. 1 ±18 L; p=0.044 | HR: 0.72 (0.46-1.12); p=0.21 | HR: 0.56 (0.21-1.45); p=0.22 at 30 days  HR: 0.52 (0.21-1.26); p=0.14 at 1 year | RR: 0.98 (0.80-1.19); p=0.81 |
| --- | --- | --- | --- | --- | --- | --- | --- | --- |
| **Primary outcome** | Efficacy: ΔCI from baseline to 72 h  Safety: Refractory CS | Area under the 72 h hs-cTnT curve | LVEF at 30 days | Composite of: In-hospital death, resuscitated CA, HT or MCS, non-fatal AMI, TIA or stroke, RRT | ΔCO as area under the curve in 3 h | Composite of: Death, resuscitated CA, another MCS at 30 days | 30 days mortality | 30 days mortality |
| **Intervention** | Epinephrine vs. Norepinephrine | MAP 80/85-100 mmHg vs. 65 mmHg in first 36 h | ECLS vs. Control | Dobutamine vs. Milrinone | Enteral bolus of Ketone Ester vs. Placebo | Immediate VA-ECMO vs. Delayed VA-ECMO if needed | VA-ECMO vs. Control | ECLS vs. Control |
| **Inclusion criteria** | AMI + CS | CA with AMI + CS | AMI + CS | CS | CS | CS | AMI + CS | AMI + CS |
| **N** | 57 | 120 | 42 | 192 | 12 | 117 | 35 | 420 |
| **Design** | Multicenter, randomized, double-blind | Multicenter, randomized, open-label | Monocentric, randomized, open-label | Monocentric, randomized, double-blind | Monocentric, randomized, double-blind | Multicenter, randomized, open-label | Multicenter, randomized, open-label | Multicenter, randomized, open-label |
| **Trial** | OMPTIMA CC^13^ | Post-hoc analysis of Neuroprotect and COMACARE^14^ | ECLS in Cardiogenic shock complicating AMI^15,16^ | DOREMI^17^ | KETO-SHOCK 1^18^ | ECMO-CS^19^ | EURO-SHOCK^20^ | ECLS-SHOCK^21^ |

| **Results** | RR: 0.83 (0.67-1.04); p=0.11 at 30 days  RR: 0.80 (0.65-0.98); p=0.027 at 6 months  RR: 0.59 (0.36-0.95); p=0.03 at 6 years | RR:1.14 (0.92-1.41); p=0.24 at 30 days  RR:1.04 (0.79-1.36); p=0.80 at 6 months | ΔCI: 0.49 ±0.46 l/min/m^2^ with Impella vs. 0.11±0.31 l/min/m^2^ with IABP; p=0.02 | Increased 28 days mortality in Dopamine group; p=0.03 | 42% with Abciximab vs. 27% usual care; p=0.24 | RR:0.96 (0.79-1.17); p=0.69 at 30 days  RR:0.99 (0.88-1.11); p=0.98 at 6 years | HR: 0.96 (0.42-2.18); p=0.92 at 30 days  HR: 1.04 (0.47-2.32); p=0.92 at 6 months  RR: 0.87 (0.47-1.59); p=0.65 at 5 years | RR: 0.83 (0.71-0.96); p=0.01 at 30 days  RR: 0.88 (0.71-0.96) for mortality at 1 year |
| --- | --- | --- | --- | --- | --- | --- | --- | --- |
| **Primary outcome** | 30 days mortality | 30 days mortality | ΔCI 30 min | 28 days mortality | Death, re-infarction, stroke or new renal failure at 30 days | 30 days mortality | 30 days mortality | Death or renal failure needing RRT at 30 days |
| **Intervention** | Early vs. Late revascularization | Tilarginine Acetate vs. placebo | Impella LP2.5 vs IABP | Dopamine vs. Norepinephrine | Upfront Abciximab vs. Optional Abciximab during PCI | IABP vs. no IABP | Impella CP vs. IABP | Culprit lesion only PCI vs. immediate multivessel PCI |
| **Inclusion criteria** | STEMI+ CS | AMI + CS | AMI + CS | Shock (cardiogenic cause subgroup) | AMI + CS | AMI + CS | STEMI + CS | STEMI + CS |
| **N** | 302 | 398 | 25 | 280 | 80 | 600 | 48 | 706 |
| **Design** | Multicenter, randomized, open-label | Multicenter, randomized, double-blind | 2 center, randomized, open-label | Multicenter, randomized, double-blind | Multicenter, randomized, open-label | Multicenter, randomized, open-label | 2 center, randomized, open-label | Multicenter, randomized, open-label |
| **Trial** | SHOCK^1,2^ | TRIUMPH^3^ | ISAR-SHOCK^4^ | SOAP II^5^ | PRAGUE-7^6^ | IABP SHOCK-II^7,8^ | IMPRESS in Severe Shock^9,10^ | CULPRIT-SHOCK^11,12^ |

**References Suppl material 1:**

Hochman JS, Sleeper LA, Webb JG, Sanborn TA, White HD, Talley JD, Buller CE, Jacobs AK, Slater JN, Col J, McKinlay SM, LeJemtel TH. Early revascularization in acute myocardial infarction complicated by cardiogenic shock. SHOCK Investigators. Should We Emergently Revascularize Occluded Coronaries for Cardiogenic Shock. N Engl J Med. 1999 Aug 26;341(9):625-34.

2 Hochman JS, Sleeper LA, Webb JG, Dzavik V, Buller CE, Aylward P, Col J, White HD; SHOCK Investigators. Early revascularization and long-term survival in cardiogenic shock complicating acute myocardial infarction. JAMA. 2006 Jun 7;295(21):2511-5.

^3^ TRIUMPH Investigators; Alexander JH, Reynolds HR, Stebbins AL, Dzavik V, Harrington RA, Van de Werf F, Hochman JS. Effect of tilarginine acetate in patients with acute myocardial infarction and cardiogenic shock: the TRIUMPH randomized controlled trial. JAMA. 2007 Apr 18;297(15):1657-66.

4 Seyfarth M, Sibbing D, Bauer I, Fröhlich G, Bott-Flügel L, Byrne R, Dirschinger J, Kastrati A, Schömig A. A randomized clinical trial to evaluate the safety and efficacy of a percutaneous left ventricular assist device versus intra-aortic balloon pumping for treatment of cardiogenic shock caused by myocardial infarction. J Am Coll Cardiol. 2008 Nov 4;52(19):1584-8.

^5^ De Backer D, Biston P, Devriendt J, Madl C, Chochrad D, Aldecoa C, Brasseur A, Defrance P, Gottignies P, Vincent JL; SOAP II Investigators. Comparison of dopamine and norepinephrine in the treatment of shock. N Engl J Med. 2010 Mar 4;362(9):779-89.

^6^ Tousek P, Rokyta R, Tesarova J, Pudil R, Belohlavek J, Stasek J, Rohac F, Widimsky P. Routine upfront abciximab versus standard periprocedural therapy in patients undergoing primary percutaneous coronary intervention for cardiogenic shock: The PRAGUE-7 Study. An open randomized multicentre study. Acute Card Care. 2011 Sep;13(3):116-22.

7 Thiele H, Zeymer U, Neumann FJ, Ferenc M, Olbrich HG, Hausleiter J, Richardt G, Hennersdorf M, Empen K, Fuernau G, Desch S, Eitel I, Hambrecht R, Fuhrmann J, Böhm M, Ebelt H, Schneider S, Schuler G, Werdan K; IABP-SHOCK II Trial Investigators. Intraaortic balloon support for myocardial infarction with cardiogenic shock. N Engl J Med. 2012 Oct 4;367(14):1287-96.

8 Thiele H, Zeymer U, Thelemann N, Neumann FJ, Hausleiter J, Abdel-Wahab M, Meyer-Saraei R, Fuernau G, Eitel I, Hambrecht R, Böhm M, Werdan K, Felix SB, Hennersdorf M, Schneider S, Ouarrak T, Desch S, de Waha-Thiele S; IABP-SHOCK II Trial (Intraaortic Balloon Pump in Cardiogenic Shock II) Investigators; IABP-SHOCK II Investigators. Intraaortic Balloon Pump in Cardiogenic Shock Complicating Acute Myocardial Infarction: Long-Term 6-Year Outcome of the Randomized IABP-SHOCK II Trial. Circulation. 2019 Jan 15;139(3):395-403.

9 Ouweneel DM, Eriksen E, Sjauw KD, van Dongen IM, Hirsch A, Packer EJ, Vis MM, Wykrzykowska JJ, Koch KT, Baan J, de Winter RJ, Piek JJ, Lagrand WK, de Mol BA, Tijssen JG, Henriques JP. Percutaneous Mechanical Circulatory Support Versus Intra-Aortic Balloon Pump in Cardiogenic Shock After Acute Myocardial Infarction. J Am Coll Cardiol. 2017 Jan 24;69(3):278-287.

^0^ Karami M, Eriksen E, Ouweneel DM, Claessen BE, Vis MM, Baan J, Beijk M, Packer EJS, Sjauw KD, Engstrom A, Vlaar A, Lagrand WK, Henriques JPS. Long-term 5-year outcome of the randomized IMPRESS in severe shock trial: percutaneous mechanical circulatory support vs. intra-aortic balloon pump in cardiogenic shock after acute myocardial infarction. Eur Heart J Acute Cardiovasc Care. 2021 Dec 6;10(9):1009-1015.

^11^ Thiele H, Akin I, Sandri M, Fuernau G, de Waha S, Meyer-Saraei R, Nordbeck P, Geisler T, Landmesser U, Skurk C, Fach A, Lapp H, Piek JJ, Noc M, Goslar T, Felix SB, Maier LS, Stepinska J, Oldroyd K, Serpytis P, Montalescot G, Barthelemy O, Huber K, Windecker S, Savonitto S, Torremante P, Vrints C, Schneider S, Desch S, Zeymer U; CULPRIT-SHOCK Investigators. PCI Strategies in Patients with Acute Myocardial Infarction and Cardiogenic Shock. N Engl J Med. 2017 Dec 21;377(25):2419-2432.

^2^ Thiele H, Akin I, Sandri M, de Waha-Thiele S, Meyer-Saraei R, Fuernau G, Eitel I, Nordbeck P, Geisler T, Landmesser U, Skurk C, Fach A, Jobs A, Lapp H, Piek JJ, Noc M, Goslar T, Felix SB, Maier LS, Stepinska J, Oldroyd K, Serpytis P, Montalescot G, Barthelemy O, Huber K, Windecker S, Hunziker L, Savonitto S, Torremante P, Vrints C, Schneider S, Zeymer U, Desch S; CULPRIT-SHOCK Investigators. One-Year Outcomes after PCI Strategies in Cardiogenic Shock. N Engl J Med. 2018 Nov 1;379(18):1699-1710.

^13^ Levy B, Clere-Jehl R, Legras A, Morichau-Beauchant T, Leone M, Frederique G, Quenot JP, Kimmoun A, Cariou A, Lassus J, Harjola VP, Meziani F, Louis G, Rossignol P, Duarte K, Girerd N, Mebazaa A, Vignon P; Collaborators. Epinephrine Versus Norepinephrine for Cardiogenic Shock After Acute Myocardial Infarction. J Am Coll Cardiol. 2018 Jul 10;72(2):173-182.

^14^ Ameloot K, Jakkula P, Hästbacka J, Reinikainen M, Pettilä V, Loisa P, Tiainen M, Bendel S, Birkelund T, Belmans A, Palmers PJ, Bogaerts E, Lemmens R, De Deyne C, Ferdinande B, Dupont M, Janssens S, Dens J, Skrifvars MB. Optimum Blood Pressure in Patients With Shock After Acute Myocardial Infarction and Cardiac Arrest. J Am Coll Cardiol. 2020 Aug 18;76(7):812-824.

^15^ Brunner S, Guenther SPW, Lackermair K, Peterss S, Orban M, Boulesteix AL, Michel S, Hausleiter J, Massberg S, Hagl C. Extracorporeal Life Support in Cardiogenic Shock Complicating Acute Myocardial Infarction. J Am Coll Cardiol. 2019 May 14;73(18):2355-2357.

^16^ Lackermair K, Brunner S, Orban M, Peterss S, Orban M, Theiss HD, Huber BC, Juchem G, Born F, Boulesteix AL, Bauer A, Pichlmaier M, Hausleiter J, Massberg S, Hagl C, Guenther SPW. Outcome of patients treated with extracorporeal life support in cardiogenic shock complicating acute myocardial infarction: 1-year result from the ECLS-Shock study. Clin Res Cardiol. 2021 Sep;110(9):1412-1420.

^17^ Mathew R, Di Santo P, Jung RG, Marbach JA, Hutson J, Simard T, Ramirez FD, Harnett DT, Merdad A, Almufleh A, Weng W, Abdel-Razek O, Fernando SM, Kyeremanteng K, Bernick J, Wells GA, Chan V, Froeschl M, Labinaz M, Le May MR, Russo JJ, Hibbert B. Milrinone as Compared with Dobutamine in the Treatment of Cardiogenic Shock. N Engl J Med. 2021 Aug 5;385(6):516-525.

^18^ Berg-Hansen K, Christensen KH, Gopalasingam N, Nielsen R, Eiskjaer H, Moller N, Birkelund T, Christensen S, Wiggers H. Beneficial effects of ketone ester in patients with cardiogenic shock. A randomized, controlled, double-blind trial. JACC Heart Fail. 2023 Oct;11(10):1337-1347.

^19^ Ostadal P, Rokyta R, Karasek J, Kruger A, Vondrakova D, Janotka M, Naar J, Smalcova J, Hubatova M, Hromadka M, Volovar S, Seyfrydova M, Jarkovsky J, Svoboda M, Linhart A, Belohlavek J; ECMO-CS Investigators. Extracorporeal Membrane Oxygenation in the Therapy of Cardiogenic Shock: Results of the ECMO-CS Randomized Clinical Trial. Circulation. 2023 Feb 7;147(6):454-464.

^20^ Banning AS, Sabaté M, Orban M, Gracey J, López-Sobrino T, Massberg S, Kastrati A, Bogaerts K, Adriaenssens T, Berry C, Erglis A, Haine S, Myrmel T, Patel S, Buera I, Sionis A, Vilalta V, Yusuff H, Vrints C, Adlam D, Flather M, Gershlick AH. Venoarterial extracorporeal membrane oxygenation or standard care in patients with cardiogenic shock complicating acute myocardial infarction: the multicentre, randomised EURO SHOCK trial. EuroIntervention. 2023 Aug 21;19(6):482-492.

^21^ Thiele H, Zeymer U, Akin I, Behnes M, Rassaf T, Mahabadi AA, Lehmann R, Eitel I, Graf T, Seidler T, Schuster A, Skurk C, Duerschmied D, Clemmensen P, Hennersdorf M, Fichtlscherer S, Voigt I, Seyfarth M, John S, Ewen S, Linke A, Tigges E, Nordbeck P, Bruch L, Jung C, Franz J, Lauten P, Goslar T, Feistritzer HJ, Pöss J, Kirchhof E, Ouarrak T, Schneider S, Desch S, Freund A; ECLS-SHOCK Investigators. Extracorporeal Life Support in Infarct-Related Cardiogenic Shock. N Engl J Med. 2023 Oct 5;389(14):1286-1297.
