# Supplementary material for: Hemodynamic management of cardiogenic shock in the intensive care unit
Source: J Heart Lung Transplant. Author manuscript; Available in PMC 2025 Jul 1. (PMC11148863; doi:10.1016/j.healun.2024.03.009)
Supplement: Supp Material 2 [file NIHMS1990686-supplement-Supp_Material_2.docx]

Supplementary Material

| **Phase** | **Description** |
| --- | --- |
| **Recognize and Rescue** | Recognition is emphasized, as earlier intervention and support may be associated with better clinical outcomes.  Rescue phase varies significantly depending on the underlying etiology and clinical presentation, such as SCAI stage and arrhythmias.  Monitoring may be limited (eg: arterial blood pressure, oxygen saturation, rhythm monitoring and capnography).  Hemodynamic phenotyping may be limited to clinical assessment and bedside echocardiography.  The objective of the Rescue phase is simply to achieve hemodynamic parameters that are compatible with immediate survival (MAP 60-65mmHg). In effect, converting SCAI stage D/E or INTERMACS ½ into SCAI stage C/D or INTERMACS 2/3.  In patients with SCAI stage C/D CS, the Rescue phase may be brief. |
| **Optimization** | The objectives of the Optimization phase include:   - Achieve arterial BP target (eg: 65-70mmHg) - Achieve oxygen delivery target (eg: >300-400ml/min/m^2^)   Additional invasive hemodynamic assessment (eg: pulmonary artery catheter) may be used to complement clinical, echocardiographic and point-of-care tests to (i) improve phenotyping of CS; and (ii) assess hemodynamic response to treatment.  Hemodynamic management is tailored to the CS phenotype, including titration of vasoactive drugs and deployment/escalation of MCS, to achieve hemodynamic response.  Hemodynamic targets may be revised depending on the clinical response.  Optimization should also include non-hemodynamic management, such as:   - Correction of MCS-related complications (eg: bleeding, limb ischemia). - Titration of mechanical ventilation to minimize ventilator-induced lung injury. - Correction of metabolic derangements (eg: renal support and hyperglycemia). - Management of coagulopathy and bleeding. |
| **Stabilization** | Stabilization is characterized by sustained improvement in organ function and in most cases, resolution of hyperlactatemia. Complications related to critical illness or MCS should have an improving trajectory.  The objective of the Optimization phase is to plan/prepare for Exit Therapy.  MCS may be down-titrated to assess ‘readiness for weaning’.  Selected patients may be activated on the wait list for heart transplantation, with specific emphasis on rehabilitation and nutritional state in preparation for transplantation.  Fluid overload and coagulopathy should be corrected in preparation for durable LVAD. |
| **De-Escalation and Exit Therapy** | De-escalation and liberation from vasoactive drugs and MCS are the objective of this phase.  Liberation from temporary MCS and vasoactive drugs necessitates either sufficient cardiac recovery (with or without interventional or surgical procedures) or, in the absence of sufficient recovery, a ‘heart replacement therapy’.  The latter usually implies either heart transplantation or durable LVAD.  The approach to Exit Therapy should be planned, including the potential pre-emptive approach to MCS such as VA ECMO for post-transplant graft dysfunction or temporary right ventricular assist device support for durable LVAD. |
