# Supplementary material for: Hemodynamic management of cardiogenic shock in the intensive care unit
Source: J Heart Lung Transplant. Author manuscript; Available in PMC 2025 Jul 1. (PMC11148863; doi:10.1016/j.healun.2024.03.009)
Supplement: Supp Material 3 [file NIHMS1990686-supplement-Supp_Material_3.docx]

**Supplementary Material 3**

The six-point plan in sepsis (Barcelona Declaration) adapted for CS.

| Awareness | Increase awareness of health care professionals, governments, health and funding agencies, and the public of the high frequency and mortality associated with CS |
| --- | --- |
| Diagnosis | Improve the early and accurate diagnosis of CS by developing a clear and clinically relevant definition and disseminating it to our peers |
| Treatment | Increase the use of appropriate treatments and interventions by disseminating the range of care options and urging their timely use |
| Education | Encourage the education of all health care professionals who manage patients with CS by providing leadership, support and information to them about all aspects of CS management, including diagnosis, treatments and interventions, and standards of care |
| Counselling | Provide a framework for improving and accelerating access to post-ICU care and counselling for patients with CS |
| Referral | Recognize the need for clear referral guidelines that are accepted and adopted at a local level in all countries by initiating the development of global guidelines |
