# Supplementary material for: Hemodynamic management of cardiogenic shock in the intensive care unit
Source: J Heart Lung Transplant. Author manuscript; Available in PMC 2025 Jul 1. (PMC11148863; doi:10.1016/j.healun.2024.03.009)

**Supplementary material 4**

A simplified OODA loop initially described by Colonel Boyd (pilot turned military strategist). This process of Observation – Orientation – Decision – Action with the feedback loop has been further developed in the context of military strategy and business management, but is equally adaptable to clinical practice, hypothesis testing and research.


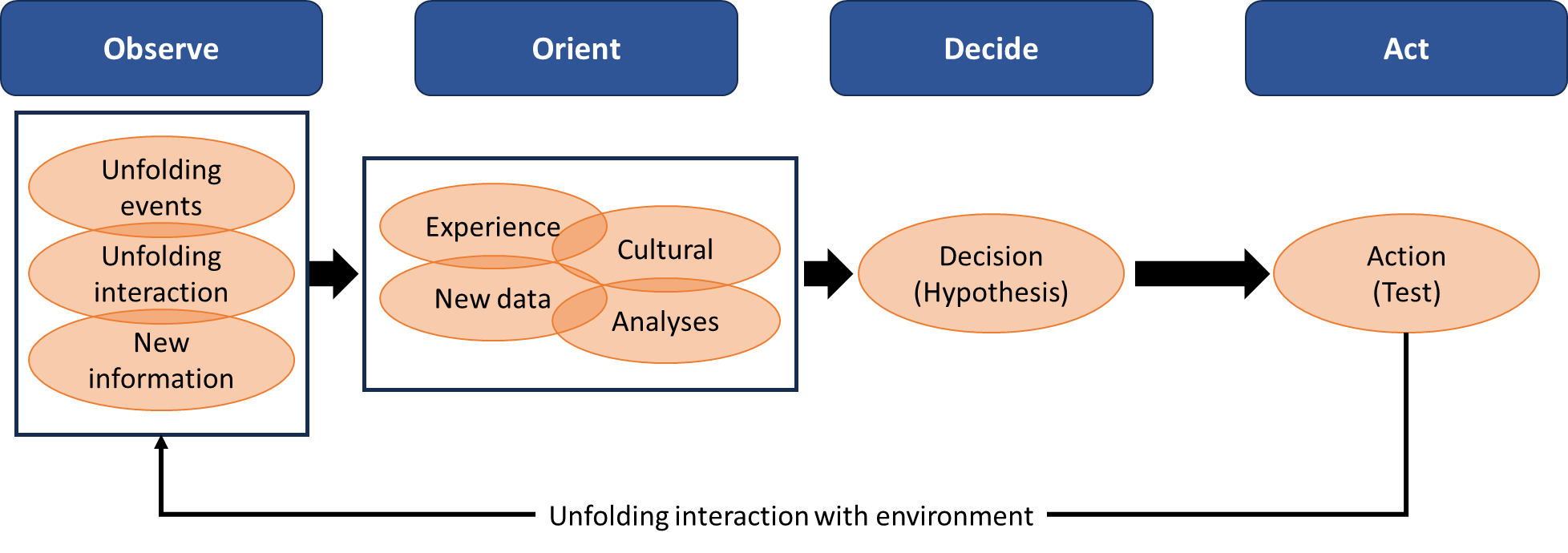

Supplement: Supp Material 4 [file NIHMS1990686-supplement-Supp_Material_4.docx]
